# Thermosetting Resin for Plug and Abandonment of Oil Wells with Reduced Environmental Impact

**DOI:** 10.3390/polym17020212

**Published:** 2025-01-16

**Authors:** Maria Echarri-Giacchi, Christian Husum Frederiksen, Lars Michael Skjolding, Anne Ladegaard Skov, Magdalena Skowyra

**Affiliations:** 1 Danish Polymer Centre, Department of Chemical and Biochemical Engineering, Technical University of Denmark, 2800 Kgs. Lyngby, Denmark; maecgi@kt.dtu.dk (M.E.-G.); al@kt.dtu.dk (A.L.S.); 2 Danish Offshore Technology Centre, Technical University of Denmark, 2800 Kgs. Lyngby, Denmark; chfred@dtu.dk; 3 Department of Environmental and Resource Engineering, Technical University of Denmark, 2800 Kgs. Lyngby, Denmark; lams@dtu.dk

**Keywords:** polymer, resin, plug and abandonment, oil wells

## Abstract

Plug and abandonment of offshore oil wells is a costly and time-consuming process, yet it is necessary for the ever-increasing number of mature fields in the region of the Danish North Sea, as well as globally. Current practices ensuring durable solutions for the complete zonal isolation of oil wells have a large environmental impact. This paper proposes a novel resin that could be mixed on the platform and pumped into the tubing in a liquid state. The increased temperature inside the oil well initiates the cross-linking reaction of the liquid resin, creating a solid and impermeable barrier. The liquid resin is thermally stable up to 180 °C and can be handled for up to 20 h at room temperature, preventing setting before intended while decreasing environmental impact. The solid resin has a compressive strength of 54 MPa and a steel adhesion strength of 6.27 MPa, highlighting its ability to withstand extreme downhole conditions.

## 1. Introduction

An oil well, after reaching the end of its life cycle, needs to be permanently plugged and abandoned (P&A) to prevent the flow of hazardous fluids into the marine environment, groundwater, ground, or atmosphere [1]. P&A operations received an increased focus in recent years, mainly due to the accumulation of old offshore wells present in mature areas, such as in the North Sea [2,3]. The traditional P&A process can be costly and time-consuming, contributing up to 25% of the total cost of offshore drilling exploration wells [1]. Decommissioning activities in the North Sea alone are estimated to reach GBP 3 billion per year in the coming years [4]. As a result, novel P&A technology that is cost-efficient and does not compromise the scope of current operations is a necessity.

Cement is the most extensively used material for oil-well isolation [1]. However, the harsh conditions present inside oil wells make them susceptible to failure and might compromise the well’s integrity [5]. This can facilitate the migration of well fluids, causing possible environmental damage, financial losses, and ultimately resulting in the need for remedial operations. In most cases, squeezing cement is used to repair the leakage paths [6]. Yet, cement presents a poor ability to penetrate small voids and cracks due to the presence of particles in the slurry, which complicates the successful repair of the damaged plug [7]. Polymer resins are also receiving significant attention as materials for remedial operations [8,9,10,11,12,13,14]. An example of that is the work by Milad et al. [8], who developed an epoxy resin with an extraordinary ability to penetrate through tight channels. The sealant is easily injected, compared to cement, and can withstand pressures up to 1000 psi after being cured inside failed cement channels.

Despite the progress in remedial operations, there is still a demand for alternatives to cement which can effectively isolate oil wells. In this context, recent studies have demonstrated the potential of polymer materials to achieve complete zonal isolation of oil wells, including either blending them with cement [15,16,17] or fully replacing cement-based barriers [18,19,20,21,22,23,24,25,26]. Keese et al. [18] synthesized and tested a novel flexible thermosetting polymer in a yard trial as an alternative to Portland cement, which showed high mechanical strength and chemical inertness. Kamali et al. [19] compared the rheological behavior and mechanical properties under downhole conditions in an expansive cement, a pozzolanic slurry, a geopolymer, and a thermosetting polymer. The latter showed higher flexibility and compressive strength than other tested materials. Recently, de Carvalho Balaban et al. [20] developed a gel with improved thermal stability, consisting of hydrolyzed polyacrylamide and polyethyleneimine reinforced with glycerol for temporary abandonment of oil wells. Looni et al. [21] investigated a new diglycidylether of bisphenol A epoxy resin for high-temperature applications by curing it with aromatic amines, which slightly improved the thermal stability of the material, as compared to the one cured with aliphatic amines.

However, as of today, one of the major gaps in the current research is the lack of studies on thermally stable polymer materials for oil well P&A, with the ability to fully isolate oil wells while also adhering to the European Chemical Strategy suggesting a zero-pollution ambition. De Stefano et al. [25] presented a polymer/monomer composite formed by thermally activated free radical polymerization reaction by using a moderately hazardous composition. However, their polymer system had restricted sealing capability, with only 51 psi of applied pressure being withstood in laboratory testing.

In this study, a novel resin for the plugging of oil wells is presented. The aim of this study is to develop a liquid resin that can be pumped directly into the oil well and cross-linked in the downhole conditions, securely isolating the oil well fluids. Additionally, the objective of this research is to design a resin based on the concept of Safe-and-Sustainable-by-Design (SSbD). This concept inherently requires fulfillment of functionality properties, such as transport safety, ease of mixing, and the ability to solidify under high temperatures, resulting in a stable resin plug of a three-dimensional network while ensuring environmental safety. Thus, in this study, the novel material’s morphological, rheological, thermal, adhesion, and mechanical properties were evaluated, and environmental hazard identification was assessed.

## 2. Materials and Methods

### 2.1. Materials

The poly(vinylidene fluoride) (PVDF, CAS: 24937-79-9, M_w_~534,000 g/mol), pentaerythritol tetraacrylate (PETA, CAS: 4986-89-4), potassium hydroxide (CAS: 1310-58-3), and potassium persulfate (PP, CAS: 7727-21-1) were purchased from Merck KGaA, Darmstadt, Germany. The ethanol (≥99.8%, CAS: 64-17-5) was purchased from VWR International, Søborg, Denmark. Hydrophobic pyrogenic silica HDK H2000 (CAS: 68909-20-6) was kindly offered by Wacker Chemie AG, Munich, Germany.

The functionalized PVDF polymer (fPVDF) was obtained by a dehydrofluorination reaction of PVDF [27,28], following the reaction scheme presented in Figure S1. An amount of 4 wt.% PVDF powder was immersed in a 5 M alcoholic potassium hydroxide solution (a mixture of 5% deionized water and 95% absolute ethanol) and heated at 60 °C for 60 min to generate unsaturated carbon−carbon double bonds. The alkali-treated PVDF powder was collected by gravity filtration, rinsed with deionized water until the filtrate was neutral, and then vacuum-dried at 60 °C for 24 h. The successful PVDF functionalization reaction was confirmed by the presence of a double bond peak on the FTIR spectra (~1600 cm^−1^), as well as by the observation of powder discoloration from white to brown. The FTIR spectra and the appearance of both the functionalized PVDF and PVDF powders can be seen in the Supplementary Information, Figures S2 and S3. Figure S2 shows no changes to C-F stretching (1180 cm^−1^) or C-F bending (830 cm^−1^) peaks after the functionalization reaction, which may be due to the lack of sensitivity of the FTIR equipment.

### 2.2. Fabrication

#### 2.2.1. Preparation of the Liquid Resin

The liquid resin was obtained by mixing fPVDF polymer with PETA cross-linker (mixing ratio of 1.1:3, respectively), together with 0.5–10 wt.% of potassium persulfate initiator and 1 wt.% of HDK H2000 additive. The liquid resin was mixed in a plastic container using a Speed Mixer DAC150.1 FVZ (Hauschild Engineering, Hamm, Germany), starting at 500 rpm for 5 s and increasing the rotations to 2500 rpm for 1.5 min, leaving a homogenous, dark liquid solution ready for the cross-linking reaction. The homogeneity of the liquid resin was assessed by visual inspection under a microscope, see Figure S4.

#### 2.2.2. Preparation of the Solid Resin

**Cross-linking in steel molds**

For material characterization purposes, solid cuboid samples were obtained by cross-linking the liquid resin in a stainless steel cuboid mold with dimensions of 30 × 30 × 2 mm. The top of the mold was covered with aluminum foil and placed in the oven at 90 °C for 24 h. A schematic of a possible chemical reaction taking place during the cross-linking reaction is shown in Figure S5.

**Cross-linking to form a solid resin plug**

A steel tube was used to mimic the geometry of oil well tubing (typically carbon steel and 10 cm in inner diameter) on a laboratory scale. A volume of 30 mL of the liquid resin was poured down into a tightly closed steel tube (Sømløs hydraulikrør ståal 37 fosfateret, Ahlsell Denmark AS, Vallensbæk, Denmark; 30 cm long, 18 mm outer diameter). After that, the top of the tube was tightly closed, and the setup was heated to 90 °C for 24 h using a specially designed heating jacket (Winkler AG, Heidelberg, Germany) to allow the resin to cross-link.

A cylindrical stainless steel mold of 18 mm in diameter and 50 mm in length was used to prepare the samples for compressive strength testing. The inner walls were sprayed with a release agent (Kema Olie CS-1300, ITW, Silkeborg, Denmark) to prevent the resin from adhering to the steel surface. The liquid resin was poured down into the steel tubing and, similarly, heated in the oven for 24 h at 90 °C.

### 2.3. Characterization Techniques

#### 2.3.1. Density

The density of the liquid resin was measured at room temperature by filling a flask of a known volume and weighing the liquid resin. The density was calculated using Equation (1):

(1) $$\rho =\frac{m}{V}$$

where *ρ* is the density of a liquid (g/cm^3^), *m* is the weight of the liquid (g), and *V* is the volume of the flask (cm^3^).

#### 2.3.2. Miscibility

The miscibility between the liquid resin and deionized water/crude oil was assessed by pouring 0.1 mL of the liquid resin into 5 mL of water/oil at room temperature. The miscibility of the mixtures was evaluated by visual inspection as the absence of layer separation (miscible) or the presence of separated layers (immiscible) upon mixing, as shown in Figure S6.

#### 2.3.3. Hardness

The hardness of the solid resin was estimated using a Shore A durometer (AD-300, Checkline Europe, Enschede, The Netherlands) on the samples prepared using a stainless steel cuboid mold. Five measurements for each sample were carried out, each with a time duration of 15 s, in accordance with ASTM D2250-15 [29].

#### 2.3.4. Environmental Hazard Identification of Chemicals

The environmental hazard identification was carried out following standardized guidelines, in accordance with ECHA and OSPAR for classical environmental impacts parameters, such as ecotoxicity, bioaccumulation, and biodegradability [30]. Ecotoxicity was tested with three aquatic organisms covering both marine (*Aliivibrio fischeri*) and freshwater species (*Raphidocelis subcapitata* and *Daphnia magna*) across trophic levels.

The ecotoxicological responses (luminescence decrease, growth inhibition, and immobilization) were plotted in GraphPad Prism (v. 10.2.2.) to estimate EC-values, concentration–response curves, and their corresponding 95% confidence intervals using non-linear curve fitting with a four-parameter logistic function. Detailed information about ecotoxicological tests is described in the Supplementary Information.

The testing for biodegradability was carried out using the OECD 301F standard with a few modifications, described in the Supplementary Information.

The bioaccumulation potential was estimated with a property-to-property estimation based on the octanol-water portioning coefficient using the Danish QSAR database [31].

#### 2.3.5. Imaging Methods

The surface morphology of the solid resin with different initiator concentrations was visually evaluated using a digital microscope, Keyence VHX-6000 (Keyence International, Mechelen, Belgium), with a VH-Z20T lens.

Computed Tomography (CT) imaging of the solid resin plug inside a steel tube was performed to evaluate the cross-linking process inside the tube and identify any voids or defects. CT images were obtained using a Nikon XT H 225 ST (Nikon Corporation, Leuven, Belgium). The electron beam was adjusted to 136 µA and 220 kV. The X-ray target was composed of Tungsten (W) with a 0.03937 Sn filter. The exposure time employed for each image was 1 s. A total of 1750 projections were obtained (1 frame/project) and a 2 × 2 binning configuration was used for image quality optimization.

#### 2.3.6. Water Pressure Resistance

**Figure 1:**
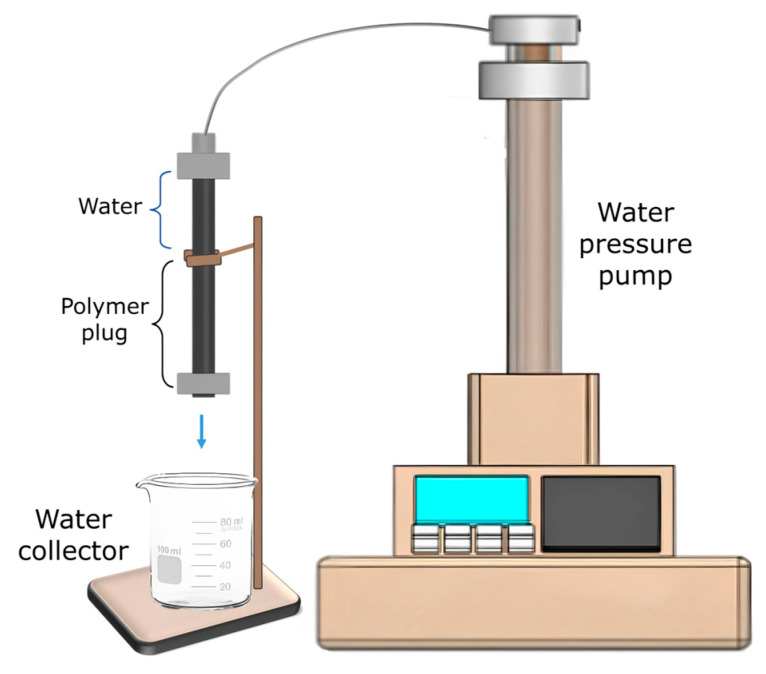
Schematic of the water pressure resistance test setup.

The resistance of the solid resin plug to applied water pressure was tested in a laboratory-scale setup using a water pressure pump (Isco D-Series Pump, Teledyne, Lincoln, NE, USA), as presented schematically in Figure 1. A steel tube plugged with the resin was placed in a vertical direction and attached to the pressure pump at the top. The bottom of the tube was opened during the test and a water collector was placed below. During the test, the pressure was gradually applied (intervals of 70 psi) up to 2175 psi or until observation of water leakage. A successful test was characterized by no water leakage through the resin plug and resin plug/steel interface at the applied pressure.

#### 2.3.7. Fourier Transform Infrared Spectroscopy (FTIR)

FTIR spectroscopy of the PVDF, functionalized PVDF, and cross-linked solid resin was conducted using an attenuated total reflectance FTIR spectrometer (Nicolet iS50, Thermo Fisher Scientific, Waltham, MA, USA). The spectral range was measured between 400 and 4000 cm^−1^, with a resolution of 4 cm^−1^, and 32 scans averaged.

#### 2.3.8. Thermogravimetric Analysis

To establish the thermal stability of the solid resin, thermogravimetric analysis (TGA) was performed. The change in the sample mass as a function of temperature was measured on a Discovery TGA (TA Instruments, New Castle, DE, USA). A total of 10 mg of the solid resin was placed in a platinum pan and heated in a nitrogen atmosphere from room temperature to 900 °C. The readout of the sample weight versus temperature was performed every 0.01 s. The results were analyzed by TRIOS software (version 5.7.0.56, TA Instruments, New Castle, DE, USA). The samples containing 0.5 wt.%, 1 wt.%, and 10 wt.% of the initiator were analyzed. The accuracy of the weight determination was <±0.1% of a measured value.

#### 2.3.9. Rheology

The rheological and viscoelastic properties of the liquid resin were tested using a rheometer (DHR-1, TA Instruments, New Castle, DE, USA) with a 20 mm parallel plate (Peltier plate steel 106669), a 1 mm gap of the sample’s thickness, 1% strain, and a frequency of oscillation of 0.1 Hz. The viscosity tests were run as flow sweep measurements between 20 °C and 50 °C (soak time 120 s) in a shear rate region of 0.01–1000 s^−1^. The oscillation time measurements were performed for temperatures varying between 20 °C and 90 °C, and for temperatures of 30 °C, 60 °C, and 90 °C, the experiments were extended until the storage modulus (G′) and loss modulus (G″) of the resin reached a plateau, indicating the formation of a stable network. Three replicates were tested and averaged for each experiment.

#### 2.3.10. Compressive Strength

The compressive strength of the solid, cylindrical resin plug, with a height-to-diameter ratio of 2:1, was assessed on a Universal Testing Machine (Instron model 1362, Norwood, MA, USA) mounted with a 100 kN load cell. A crosshead speed of 0.1 cm/min was used for the tests, following ASTM D695 [32]. Five replicates were tested and averaged. The compressive strength σ was calculated using Equation (2):

(2) $$\sigma =\frac{F}{A}$$

where σ is the compressive strength (MPa), *F* is the applied load (N), and *A* is the specimen cross-section area (mm^2^).

#### 2.3.11. Lap Shear Strength

The lap shear strength of the resin adhesion to stainless steel was determined by lap shear tests, according to the ASTM D1002 standard [33]. Stainless steel samples with dimensions of 25 × 100 × 1.6 mm were mechanically abraded with sandpaper (grit size 20), followed by wiping with isopropanol and evaporation for 10 min. Afterward, 0.02 g of the liquid resin was placed on 25 × 12.5 mm of the abraded surface, and another abraded stainless steel plate was placed on top. Then, the specimens were placed in the oven at 90 °C for a week. Additionally, three of the specimens were immersed in deionized water for three months and tested to evaluate the influence of water on the adhesion strength.

The lap shear strength of the resin was tested using a Universal testing machine (Instron 88R1331, Norwood, MA, USA) with a 50 kN load cell. The crosshead speed of 1.3 mm/min was used for all tests. Five replicates for each adhesive joint were tested and averaged, and the failure types were inspected visually.

## 3. Results and Discussion

### 3.1. Environmental Hazard Identification of Chemicals

The chemicals (fPVDF, PETA, PP, and silica additive) were evaluated individually with combined classifications of the OSPAR and ECHA guidelines for classification and labeling for pre-market registration [30]. Early design phase principles for environmental safety were used and their results are summarized in Table 1.

The acute toxicity was found to require no labeling, neither in the context of OSPAR guidelines nor ECHA thresholds for classification or labeling based on acute toxicity (EC_50_ < 100 mg/L) for fPVDF, PETA, and silica additive. PP was flagged as a yellow production chemical in OSPAR and as acute category 3 in the ECHA classification and labeling, however, it is used in very small quantities in the resin. None of the substances were found to be potentially bioaccumulative based on a property-by-property assessment.

The formed resin plug aims to remain durable in the oil well; therefore, the compounds should not be easily biodegradable. Thus, none of the plug constituents passed the Level 0 test for ready biodegradability. While this would require further attention for substances that could be discharged into the environment, in the context of this study, it is a requirement. Therefore, no further evaluation of the biodegradability was carried out.

Consequently, the early design phase screening did not indicate environmental hazards and offered a qualitatively better solution compared to current plug and abandonment technologies. However, if the up-scaling of the method is expected, the long-term effects should be investigated further.

### 3.2. Optimal Initiator Concentration

The initiator concentration can significantly influence the workability, reaction rate, and final morphology of the resin. In the liquid state, the resin should have a long working time at room temperature to allow mixing and pumping on the platform, and a high reaction rate at higher temperatures to cross-link once placed downhole. Moreover, to act as a sealing material, the cross-linked solid resin should be homogeneous and uniform, with no connected pores in the material volume.

#### 3.2.1. Microscopy

**Figure 2:**
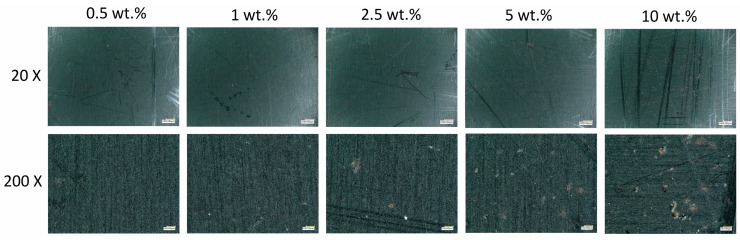
Microscopy images of the solid resin samples containing 0.5 wt.%, 1 wt.%, 2.5 wt.%, 5 wt.%, and 10 wt.% of the initiator (PP). The upper row presents a zoom-in of 20× and the lower row a zoom-in of 200×. The scale bar represents 100 µm.

The influence of the initiator concentration on the microstructure of the solid resin was evaluated using optical microscopy. The surface morphology of solid samples containing 0.5 wt.%, 1 wt.%, 2.5 wt.%, 5 wt.%, and 10 wt.% of the initiator (potassium persulfate, PP) are shown in Figure 2. At higher initiator concentrations, the samples had more irregular surfaces, with the presence of particles and a few micro-pores for the 10 wt.% sample, indicating the cross-linking reaction rate was too high. The defects are likely caused by areas of fast localized polymerization that do not allow uniform polymer chain reactions and rearrangements. At lower initiator concentrations, the samples showed smoother and more homogeneous surfaces, with no visible pores or particles, indicating an optimal cross-linking process. Therefore, a concentration lower than 2.5 wt.% of the initiator was required to obtain a uniform solid resin.

#### 3.2.2. Computed Tomography and Water Pressure Resistance

CT scanning was used to reconstruct images into a 3D model in order to analyze the internal structure of the solid resin once it had been cross-linked inside a steel tube. The goal was to obtain a resin plug free from internal defects, voids, cracks, or imperfections that could influence the sealing capability or mechanical integrity of the plug.

**Figure 3:**
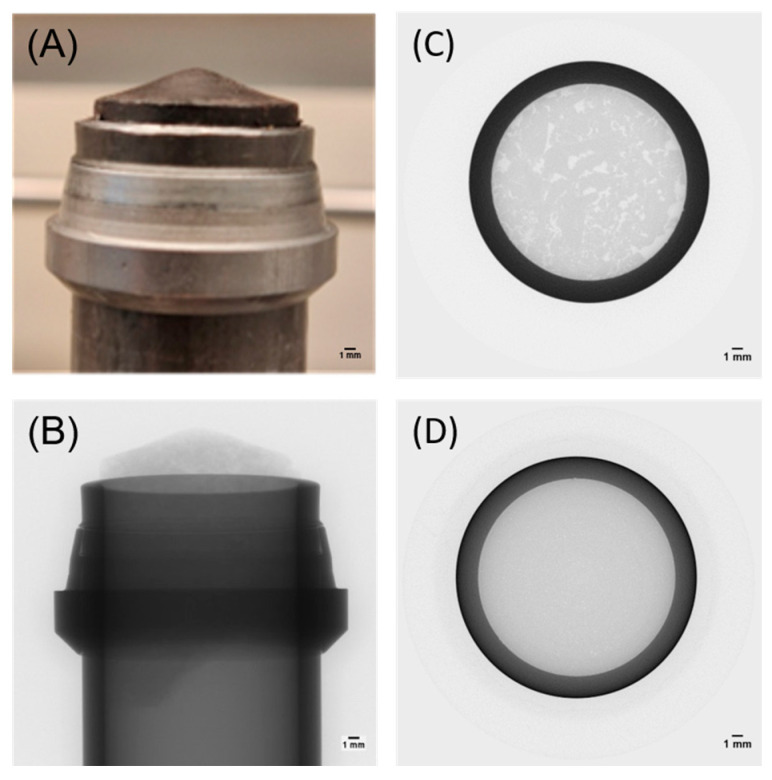
(**A**) The solid resin plug inside a steel tube, (**B**) the CT scan of (**A**), (**C**) a cross-section CT scan image of a sample with 10 wt.% initiator, and (**D**) a cross-section CT scan image of a sample with 0.5 wt.% initiator. The increased grayscale intensity corresponds to a higher density. The scale bar represents 1 mm.

Figure 3A shows the solid resin plug cross-linked inside a steel tube and Figure 3B shows its 3D visualization using CT. The high density of steel caused the initial beam to undergo attenuation and the contrast between the material and steel became lower. However, the top part of the plug (B) reflects its equivalent (A).

A more precise analysis can be performed by looking at the cross-sectional images of Figure 3B, as shown in Figure 3C,D. The resin plug (C), containing 10 wt.% of the initiator, presented a multitude of defects and voids, seen as different gray intensities in the material’s volume. Lowering the concentration of the initiator to 0.5 wt.% strongly improved the homogeneity of the sample, as seen in Figure 3D, due to the more efficient cross-linking reaction. For the 0.5 wt.% initiator sample, the resin formed a uniform plug all over the length of the steel tube, with no cracks and imperfections along the length.

To further examine the sealing capabilities of the plug, the 0.5 wt.%, 1 wt.%, and 10 wt.% initiator resin plug samples were subjected to water pressure tests, where the material was exposed to a pressurized water flow over time. The resin plug containing the 10 wt.% initiator sample could not withstand atmospheric water pressure and leaked water from the beginning of the test, due to the connected pores present in the structure, as seen in Figure 3C. Contrarily, the resin plugs containing 0.5 wt.% and 1 wt.% initiators were able to withstand atmospheric water pressure for up to 30 days, as well as water pressure of 200 psi and 115 psi, respectively. This sealing capacity improvement can be linked to a more uniform structure with fewer internal defects, voids, and cracks.

#### 3.2.3. Thermal Stability

**Figure 4:**
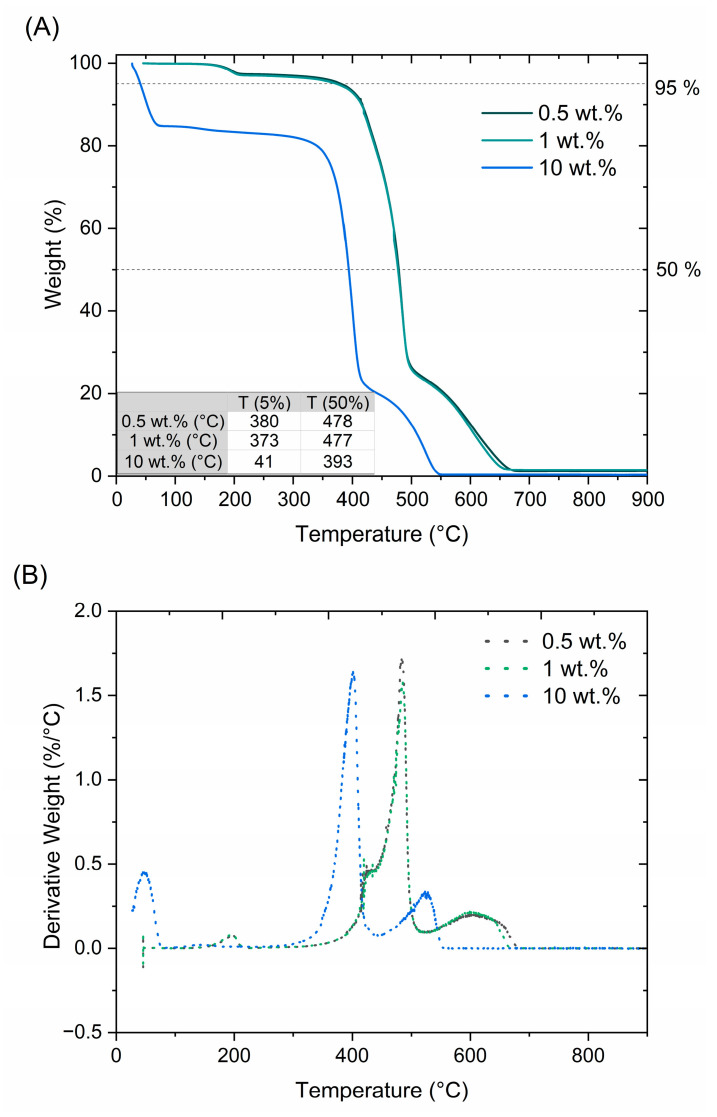
Thermal decomposition of the solid resin with 0.5 wt.%, 1 wt.%, and 10 wt.% of the initiator (PP). (**A**) TGA curves: the temperatures T (5%) and T (50%), for which 5% and 50% of the solid resin mass was decomposed, are highlighted on the TGA graph by horizontal dashed lines and summarized in the table. (**B**) Derivatives of the respective TGA curves.

The solid polymer was designed to exhibit thermal stability up to 90 °C, which is the temperature in the chosen downhole conditions. Figure 4A shows the thermal stability of the solid resin containing 0.5 wt.%, 1 wt.%, and 10 wt.% initiator, and Figure 4B shows the derivatives of the respective curves. For samples containing 0.5 wt.% and 1 wt.% of the initiator, the main degradation step was at 400–500 °C, and full decomposition was at 550–700 °C. For samples containing 10 wt.% of the initiator, the main degradation step was at 350–450 °C, while the full decomposition was at 450–550 °C.

No significant difference was observed for samples containing 0.5 wt.% and 1 wt.% of the initiator, and both were thermally stable beyond 90 °C. The first weight loss was observed at 180 °C, which can be attributed to the decomposition of the unreacted initiator [34], as well as trapped residual moisture and fPVDF decomposition, see Figure S7. The thermograms of the samples containing 2.5 wt.% and 5 wt.% of the initiator were added to the Supplementary Information, Figure S8.

In contrast, the solid resin containing 10 wt.% of the initiator had significantly lower thermal stability due to the aforementioned inefficiency of the cross-linking process (Section 3.2.1 and Section 3.2.2) and showed weight losses up to 15% in the intended temperature range of 20–90 °C. As a result, this composition was not considered for further use due to its thermal instability, as compared to samples containing 0.5 wt.% and 1 wt.% of the initiator.

### 3.3. Rheological Characterization

The liquid resin is designed to be prepared on the oil platform, by mixing all the compounds on-site. Then, the formed liquid resin is intended to be pumped down the oil-well tubing, where the cross-linking process would take place, initiated by increased temperatures. Therefore, the rheological properties of the liquid resin are crucial during the mixing, pumping, and setting of the resin.

As discussed in Section 3.2, the 0.5 wt.% initiator sample showed the most suitable characteristics for plugging and abandonment of oil wells; hence, it was further characterized.

#### 3.3.1. Viscosity of the Resin

**Figure 5:**
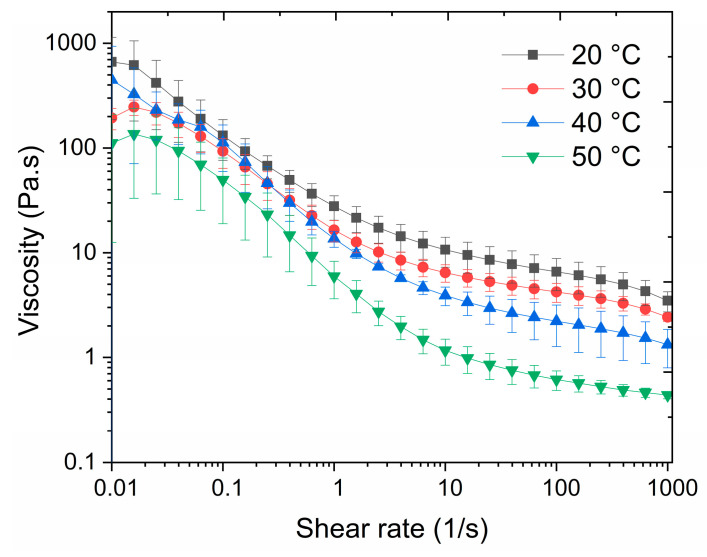
The influence of the shear rate on the viscosity of the liquid resin at temperatures between 20 °C and 50 °C. The solid curves represent the average viscosity value of three samples.

The viscosity of the liquid resin was measured at different temperatures and shear rates, as can be seen in Figure 5. For all tested temperatures, the viscosity decreased with an increase in the shearing rate, highlighting that the resin was a shear-thinning fluid. At low shear rates, the resin showed a maximum of 800 Pa·s at 20 °C. As the shear rate increased, the viscosity of the resin decreased significantly, reaching a minimum viscosity of 0.15 Pa·s at 1000 s^−1^ at 50 °C. The change in viscosity was more pronounced at higher temperatures, indicating a temperature-dependent viscosity of the resin. This behavior can be further explained by the increased mobility of molecules at higher temperatures, which results in reduced intermolecular forces and lower viscosity. Thus, under pumping conditions where high shear rates are used, and heat is generated due to friction, the liquid resin shows improved flow properties.

#### 3.3.2. Cross-Linking Time Dependence on Temperature

**Figure 6:**
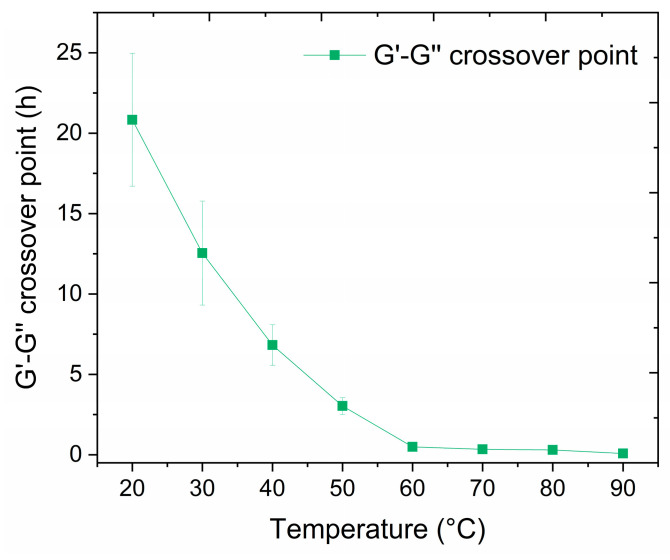
Variation in the storage (G′) and loss (G″) moduli crossover point as a function of the cross-linking temperature.

The setting time of the liquid resin and its impact on the workability of the material was evaluated at temperatures between 20 °C and 90 °C. The liquid resin must have a sufficiently long working time at room temperature (>5 h), allowing for handling and pumping it before the cross-linking reaction occurs. The transition from liquid-like to solid-like behavior of the sample was determined as a crossover point when storage modulus (G′) and loss modulus (G″) were equal. As shown in Figure 6 and Table 2, the G′-G″ crossover point of the resin at 20 °C and 30 °C showed a slow cross-linking process, with material changing to a solid-like behavior after 20.8 h and 12.5 h of the reaction, respectively. At higher temperatures, reflecting the conditions inside the oil well, the cross-linking process was accelerated, and at 90 °C, the transition from liquid-like behavior to solid-like behavior lasted only 5 min.

The temperature also affects the strength of the formed solid resin, as shown in Figure S9 and Table 2. Rheology experiments at temperatures of 30 °C, 60 °C, and 90 °C indicate that the material’s stiffness and resistance to deformation increased with the increase of the cross-linking temperature. Higher G′ values at higher temperatures could be attributed to a more effective cross-linking process, closely linked to a greater strength of the resin. The setup throughout the test resulted in the cross-linking reaction being more susceptible to oxygen [35] than during the original cross-linking inside a steel mold, leading to lower G′ values after cross-linking. Therefore, the G′ values after 72 h were only used for comparison purposes and should not be considered as absolute values.

### 3.4. Solid Polymer Plug Characterization

**Figure 7:**
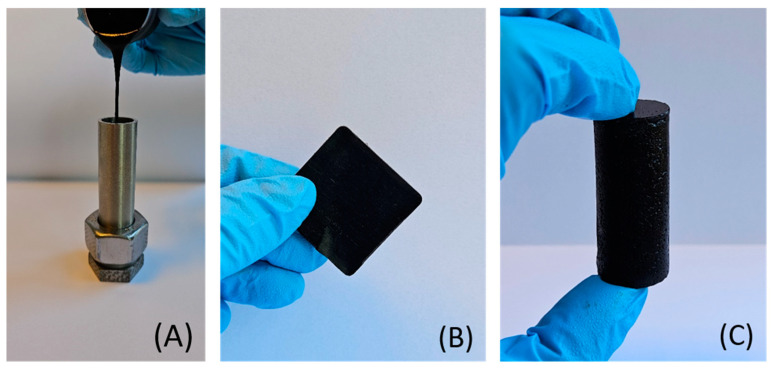
(**A**) The liquid resin being poured into a steel tube. (**B**) The solid resin after cross-linking in a square steel mold. (**C**) The solid resin plug after cross-linking in a steel tube mold.

The appearance of the liquid resin after the mixing process can be observed in Figure 7A. The resin had a dark brown color, a density of 1.33 g/cm^3^, and a viscosity of 800 Pa·s at 20 °C, see Table 3. After cross-linking at elevated temperatures, the liquid resin became solid and could adapt its shape depending on the cross-linking mold, as observed in Figure 7B,C. The solid resin showed a thermal stability up to 180 °C, a hardness of 93 ± 5 in the Shore A durometer scale range, and a compressive strength of 54 ± 4 MPa. The structural characterization of the solid resin was performed by FTIR, and the spectrum can be seen in the Supplementary Information, Figure S10. The mechanical properties of the solid resin will be discussed in the next sections. Future work will focus on the long-term stability of the polymer, resembling the methodology employed by other researchers [36,37].

#### 3.4.1. Compressive Strength

Oil well plugging material must have sufficient compressive strength to ensure effective zonal isolation of the well, where downhole pressure and mechanical stresses are present [1].

**Figure 8:**
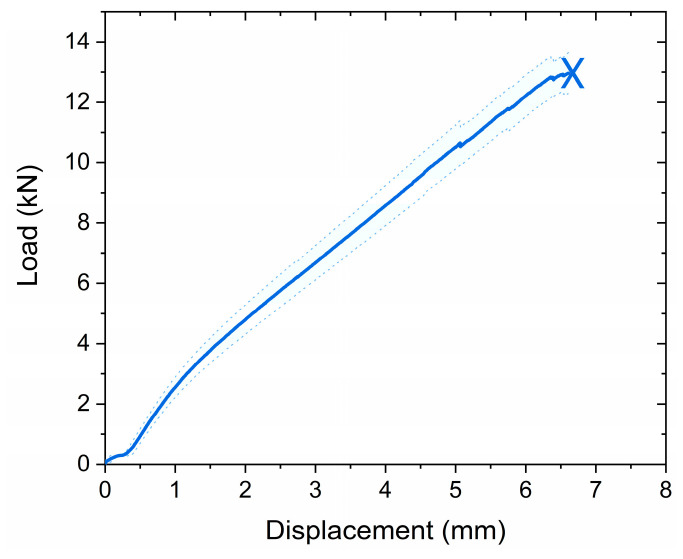
A load–displacement curve of solid resin plugs (0.5 wt.% initiator) tested during compression. The blue solid curve represents the average load value of five samples with a cross-sectional area of 2.54 cm^2^. The error bar areas are filled under the blue dotted curve.

The capacity of the solid resin plug to withstand mechanical load was tested during the compression tests. As presented in Figure 8, the cylindrical solid resin plug with a height of 35 mm and diameter of 17.5 mm was able to withstand compressive loads up to 12.9 ± 0.7 kN before failure, which resulted in a compressive strength value equal to 54 ± 4 MPa. The observed compressive strength outperformed the value of 36 MPa reported for API Class-G cement [38], commonly used for plug and abandonment of oil wells, indicating satisfactory mechanical properties to ensure well integrity.

#### 3.4.2. Lap Shear Strength

The adhesion between the steel casing and the plug is a key parameter for the successful plugging of an oil well. A high lap shear strength of the material for bonding stainless steel will ensure a durable and successful abandonment process.

**Figure 9:**
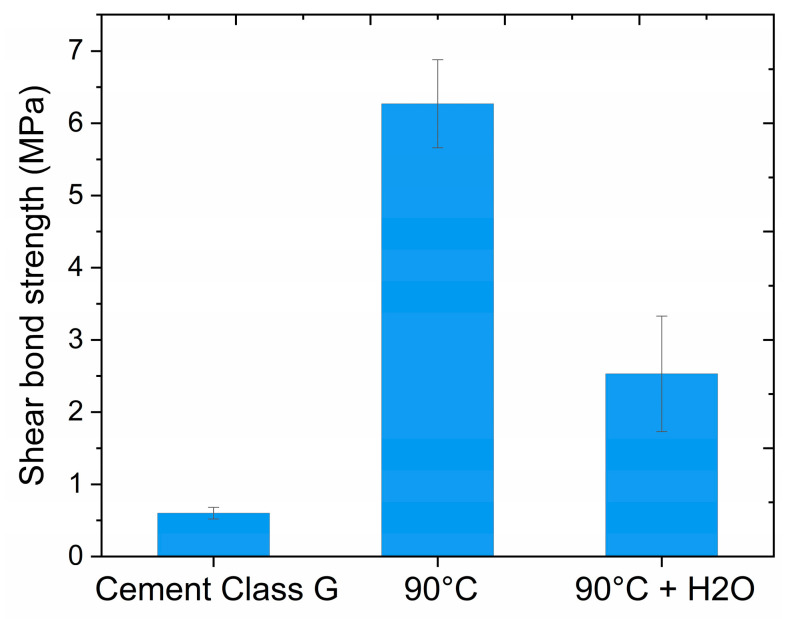
Comparison of the shear bond strength of the API class-G cement [38], resin cross-linked at 90 °C, and resin cross-linked at 90 °C and additionally kept in water for 3 months (90 °C + H_2_O). The error bars represent the standard deviation of five replicates.

The single-lap shear strength results of the stainless steel/solid resin/stainless steel plates are presented in Figure 9. The solid polymer (90 °C) showed a single-lap shear strength of 6.27 ± 0.61 MPa, with an adhesive mode of failure. Compared to API class-G cement, reported by Saasen et al. [39], the solid resin’s shear strength was more than 10 times higher, demonstrating a good ability of the material to adhere to steel casing. This strong adhesion of the resin to steel is probably due to the high concentration of acrylic moieties present in the PETA cross-linker, which can interact with the steel surface by hydrogen bonds, van der Waals forces, and chemical reactions [40]. As the plug might also be in contact with water-based fluids, the influence of water on the adhesion was evaluated by immersing the stainless steel/solid resin/stainless steel plates in deionized water for 3 months. After this period, the resin was still adhering to the steel plates, but the single-lap shear strength decreased to 2.53 ± 0.80 MPa, similarly showing an adhesive mode of failure. This result showed that the steel–resin adhesion in a wet environment is limited compared to the standard dry conditions, which could be attributed to an interfacial degradation due to mechanisms such as resin swelling, hydration layers formation, or hydrolysis of the resin chains [41]. Nevertheless, the adhesion remained stronger than reported for API class-G cement.

## 4. Conclusions

This study introduced a novel thermosetting resin as an alternative to cement for plugging and abandonment of oil wells. The material was designed to have a temperature-dependent cross-linking time and pumpable properties. The resin’s composition was adjusted in terms of the initiator concentration, with 0.5 wt.% of potassium persulfate chosen as an optimum amount based on the resin’s improved morphology, enhanced thermal stability, and reduced environmental impact. Three of the chemicals composing the resin were found to be below current thresholds for classification as acutely hazardous to the aquatic environment. In terms of functionality, the resin was found to be suitable for transportation and operation on an offshore platform. The resin components could be easily mixed and pumped down into the production casing due to a sufficiently long reaction time of 20 h at room temperature. At elevated temperatures, equivalent to those present in downhole conditions, the polymer undergoes a cross-linking reaction to form a solid resin. The liquid–solid transition accelerated to 5 min at 90 °C, and after 24 h, a homogenous and stiff material was formed. The solid resin had a high compressive strength of 54 ± 4 MPa, outperforming commonly used API class-G cement. Moreover, it was thermally stable up to 180 °C and adhered to the steel casing with a lap shear strength of 6.27 ± 0.61 MPa. While this work presented a proof-of-concept for a polymer-based alternative to cement material, future research will focus on scaling up the technology to achieve a higher Technology Readiness Level, including an assessment of its cost-effectiveness.

## Figures and Tables

**Table 1:** Environmental hazard identification of the compounds used for the preparation of the solid resin plug. EC—effect concentration. The numbers in hard brackets represent the 95% confidence interval of the concentration–response fitting.

Compound	Aquatic Toxicity (mg/L)			Potential Bioaccumulation from Kow (QSAR)	Biodegradability (Level 0)
	Microtox EC_30min_, 50 (*A. fischeri*)	Freshwater Algae EC_72h_, 50 (*R. subcapitata*)	Freshwater Crustacean EC_48h_, 50 (*D. magna*)		
fPVDF	>100	>100	>100	-	Not pass
Cross-linking agent (PETA)	>100	103 [66–140]	>100	No	Not pass
Initiator (PP)	>100	29 [20–38]	>100	No	-
Additive (Silica)	>100 *	>100 *	>100 *	No	Not pass

Symbol ‘-’ indicates that the compound was not tested for specific properties. Symbol ‘*’ indicates that only a water-accommodated fraction (aqueous phase containing dissolved and/or stable dispersions of the compound) was tested.

**Table 2:** G′-G″ crossover point of storage (G′) and loss (G″) moduli at different cross-linking temperatures. G′ at temperatures of 30 °C, 60 °C, and 90 °C after 72 h are also depicted.

Temperature								
	20 °C	30 °C	40 °C	50 °C	60 °C	70 °C	80 °C	90 °C
G′-G″ crossover point (h)	20.8 ± 4.1	12.5 ± 3.2	6.8 ± 1.3	3.0 ± 0.5	0.49 ± 0.03	0.34 ± 0.07	0.30 ± 0.03	0.08 ± 0.03
G′ after 72 h (kPa)	-	53.9	-	-	61.9	-	-	17,600

**Table 3:** General properties of the 0.5 wt.% initiator resin.

Property	Value
Density at 25 °C	1.33 g/cm^3^
Viscosity (0.1–1000 1/s, 20–50 °C)	800–0.15 Pa·s
Setting time (20–90 °C)	20.8–0.08 h
Miscibility with water and oil	No
Pumpability	Yes
Target oil-well temperature	90 °C
Thermal stability up to	180 °C
Hardness—Shore A	93 ± 5 A
Compressive strength	54 ± 4 MPa

## Data Availability

The original contributions presented in this study are included in the article/Supplementary Materials. Further inquiries can be directed to the corresponding author.

## Supplementary Materials

The following supporting information can be downloaded at: https://www.mdpi.com/article/10.3390/polym17020212/s1. Methods description: the environmental hazard identification of chemicals; Figure S1: possible reaction scheme for the dehydrofluorination of PDVF; Figure S2: FTIR spectra of PVDF and functionalized PVDF (fPVDF) after the dehydrofluorination reaction; Figure S3: PVDF powder (a) before and (b) after the dehydrofluorination reaction (fPVDF); Figure S4: Microscope image of the liquid resin after the mixing step. The scale bar represents 100 µm; Figure S5: schematic of a possible chemical reaction taking place during the cross-linking reaction between fPVDF and PETA; Figure S6: The miscibility of the liquid resin with (a) water, and (b) oil. Left column: side view, and right column: top view; Figure S7: TGA of PVDF (blue curve) and fPVDF (functionalized PVDF, black curve) powder; Figure S8: The thermal decomposition of the solid resin with 2.5 wt.% (blue solid curve) and 5 wt.% (green solid curve) of initiator (PP). The dotted lines represent derivatives of the respective curves; Figure S9: the variation of the storage (G′, blue) and loss (G″, green) moduli as a function of time for temperatures: (a) 30 °C, (b) 60 °C, and (c) 90 °C; and Figure S10: FTIR spectrum of the solid resin with 0.5 wt.% of initiator (PP).
