# Supplementary material for: Thermosetting Resin for Plug and Abandonment of Oil Wells with Reduced Environmental Impact
Source: Polymers (Basel). 2025 Jan 16;17(2):212. doi: 10.3390/polym17020212 (PMC11769116; doi:10.3390/polym17020212)
Supplement: Supplementary file 1 [file polymers-17-00212-s001.zip › polymers-3402808-supplementary.pdf]

Thermosetting resin for plug and abandonment of oil wells with reduced environmental impact

Maria Echarri-Giacchi [1], Christian Husum Frederiksen [2], Lars Michael Skjolding [3], Anne Ladegaard Skov [1], and Magdalena Skowyra [1,*]

1 Danish Polymer Centre, Department of Chemical and Biochemical Engineering, Technical University of Denmark, Kgs. Lyngby, Denmark
2 Danish Offshore Technology Centre, Technical University of Denmark, Kgs. Lyngby, Denmark
3 Department of Environmental and Resource Engineering, Technical University of Denmark, 2800 Kgs. Lyngby, Denmark

* Correspondence: magsk@dtu.dk

Environmental Hazard Identification of Chemicals

Ecotoxicity tests

The decrease in bacterial luminescence (*A. fischeri*, formerly *Vibrio fischeri*) was quantified following ISO 11358-3 with modification [1]. Freeze-dried bacteria were reconstituted in 12 mL saltwater solution (2 wt.%) and left for 20 min before initiating the experiment. A two-fold serial dilution of respectively fPVDF, PETA, PP and silica additive was prepared in the 12.5 - 200 mg/L range. The background luminescence of a 2 wt.% saltwater solution was determined using a luminometer and the experiment was initiated by adding 100 µL of bacteria to the serial dilution, resulting in a nominal exposure concentration in the range of 6.25 – 100 mg/L. Luminescence was measured after 0 and 30 min of exposure. The test was considered valid if the parallel determination of the controls did not deviate more than 3% and 30 min exposure to 3.4 mg/L 3,5-dichlorophenol caused between 20 and 80% decrease in luminescence.

Algal growth inhibition test was carried out according to OECD 201 using green algae (*R. subcapitata*, formerly *Selenastrum capricortunum* and *Pseudokirchneriella subcapitata*) obtained from NIVA (Norwegian Institute for Water Research, Oslo, Norway) [2]. Nominal exposure concentrations were prepared in the range of 6.25 – 100 mg/L using a two-fold dilution between concentrations. Exponentially growing algal cultures were inoculated at 10.000 cells/mL in 20 mL glass scintillation vials. Incubation was carried out with orbital shakers illuminated from below with fluorescent tubes with a light intensity of 100 ± 20 $\mu mol \cdot m^{-2} \cdot s^{-1}$. Biomass was quantified using acetone extraction of the algal pigments and measuring the in vitro fluorescence by excitation and emission at 430 and 670 nm respectively [3].

Acute immobilization tests with crustaceans were conducted according to OECD 202 by exposing *D. magna* neonates for 48 h to nominal exposure concentrations in the range of 6.25 – 100 mg/L in 25 mL glass beakers [4]. Approximately five organisms were exposed in each beaker. The number of immobile organisms was counted after 48 h of exposure. pH and dissolved oxygen concentrations were measured initially and at the end of the test and were within acceptable limits.

Biodegradability tests

Activated sludge was collected from Mølleåværket wastewater treatment plant (Lundtofte, Denmark) in 1L blue cap bottles. The sludge was filtered through a 500 µm sieve and aerated until use. Total suspended solids (TSS) were determined by filtering and drying 5 mL sludge and the number was used to calculate the final content of TSS in the exposures to be in the range of 20-30 mg TSS/L. The theoretical oxygen demand (ThOD) of fPVDF, PETA and PP was estimated to calculate the addition volume to reach approximately 80 mg ThOD/L in a final volume of 365 mL. Quality criteria for the test include

the oxygen uptake of the inoculum blank to be in the range of 20-30 mg O_2/L and not exceeding 60 mg O_2/L, and the pH should be in the range of 6-8.5 at the end of the test.

[1]: ISO 11348-3:2007; Water quality - Determination of inhibitory effect of water samples on the light emission of *Vibrio fischeri* (Luminescent bacteria test) - Part 3: Method using freeze-dried bacteria. International Organization for Standardization: Geneva, Switzerland, 2007.

[2]: OECD, 2011. Test No. 201: Freshwater Alga and Cyanobacteria, Growth Inhibition Test. OECD Guidelines for the Testing of Chemicals, Section 2, OECD Publishing, Paris, France, 2011. https://doi.org/10.1787/9789264069923-en.

[3]: Mayer, P.; Cuhel, R.; Nyholm, N. A simple in vitro fluorescence method for biomass measurements in algal growth inhibition tests. *Water Research*. **1997**, *31*, 2525–2531. https://doi.org/10.1016/S0043-1354(97)00084-5.

[4]: OECD, 2004. Test No. 202: Daphnia sp. Acute Immobilisation Test. OECD Guidelines for the Testing of Chemicals, Section 2, OECD Publishing, Paris, France, 2004. https://doi.org/10.1787/9789264069947-en.

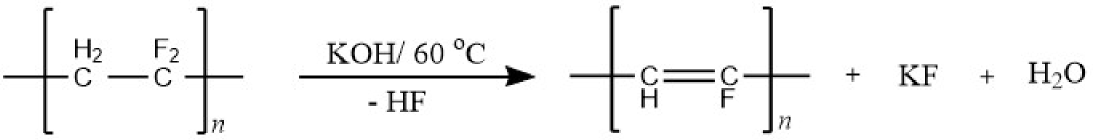

Figure S1. Possible reaction scheme for dehydrofluorination of PDVF.

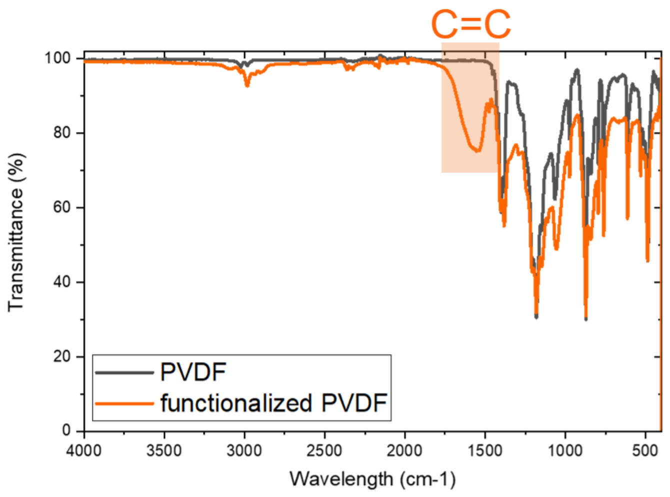

Figure S2. FTIR spectra of PVDF and functionalized PVDF (fPVDF) after the dehydrofluorination reaction.

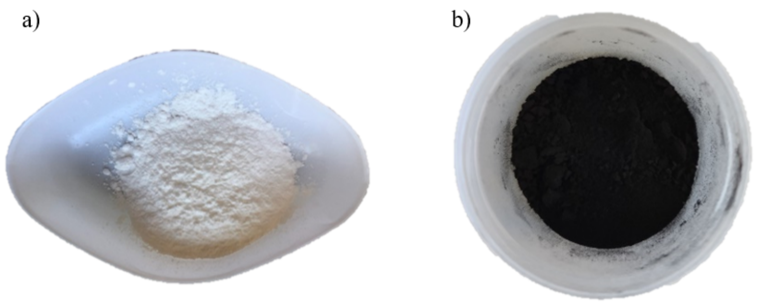

Figure S3. PVDF powder: (a) before, and (b) after the dehydrofluorination reaction (fPVDF).

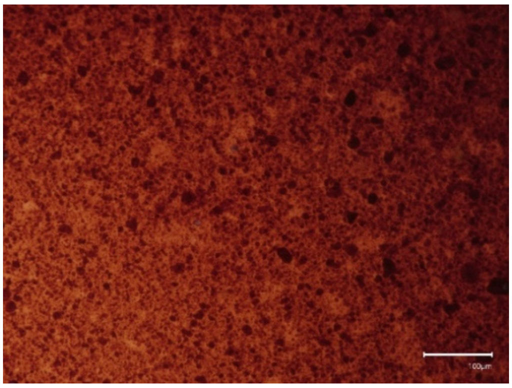

Figure S4. Microscope image of the liquid resin after the mixing step. The scale bar represents 100 μm.

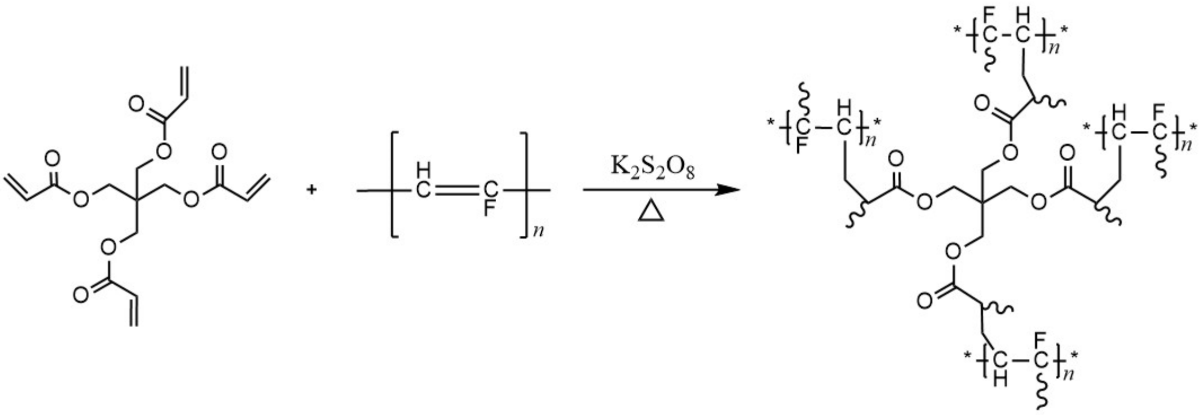

Figure S5. Schematic of a possible chemical reaction taking place during the cross-linking reaction between fPVDF and PETA.

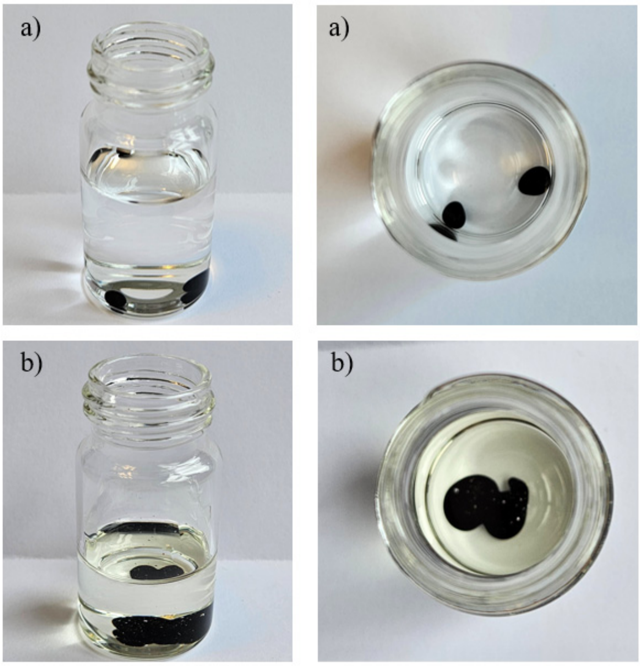

Figure S6. Miscibility of the liquid resin with a) water, and b) oil. Left column: side view; right column: top view.

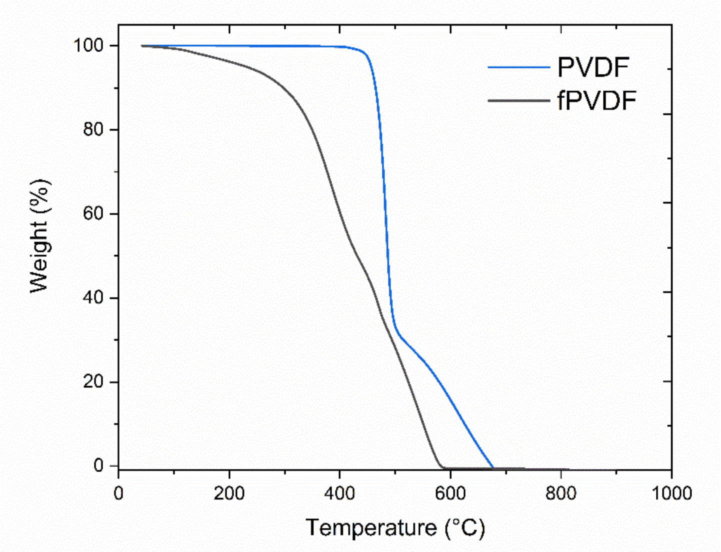

Figure S7. TGA of PVDF (blue curve) and fPVDF (functionalized PVDF, black curve) powder.

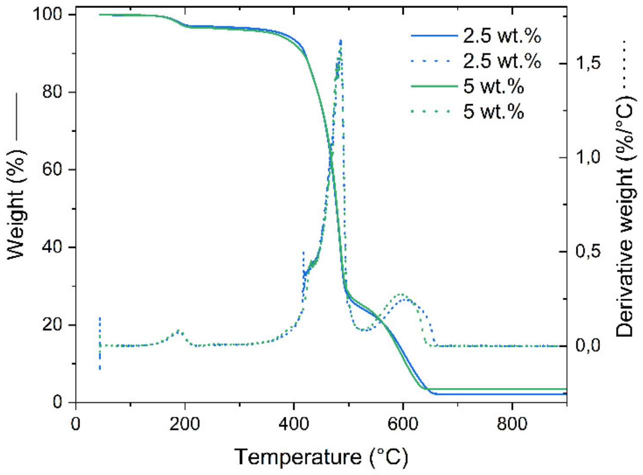

Figure S8. Thermal decomposition of solid resin with 2.5 wt.% (blue solid curve) and 5 wt.% (green solid curve) of initiator (PP). Dotted lines represent derivatives of the respective curves.

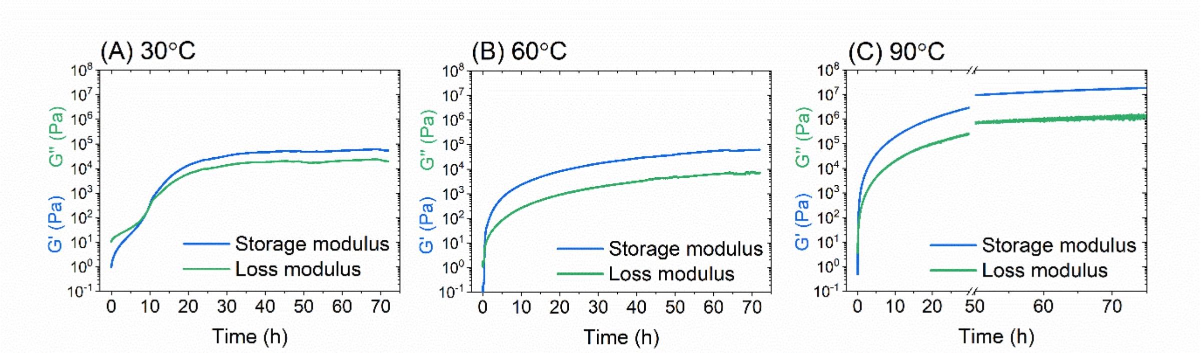

Figure S9. Variation of the storage (G′, blue) and loss (G″, green) moduli as a function of time for temperatures: (a) 30 °C, (b) 60 °C, and (c) 90 °C.

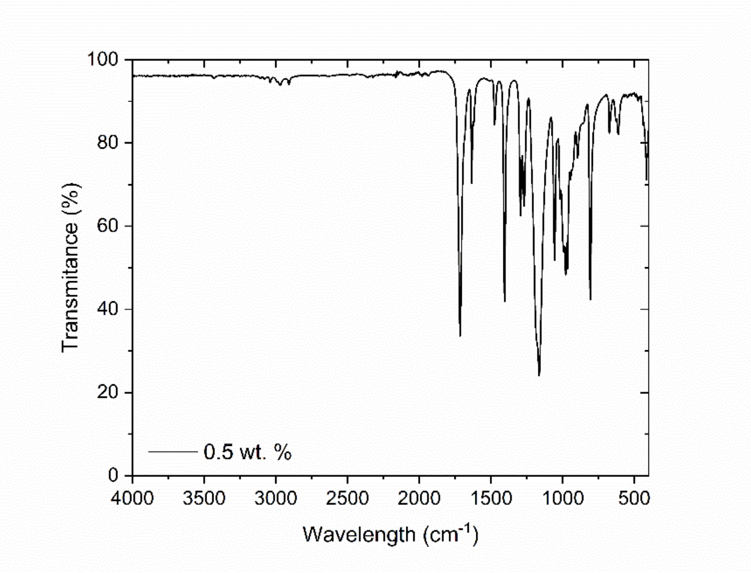

Figure S10. FTIR spectrum of the solid resin with 0.5 wt.% of initiator (PP).
